# A quantitative engineering study of ecosystem robustness using thermodynamic power cycles as case studies

**DOI:** 10.1371/journal.pone.0226993

**Published:** 2019-12-31

**Authors:** Varuneswara Panyam, Astrid Layton

**Affiliations:** J. Mike Walker ‘66 Department of Mechanical Engineering, Texas A&M University, College Station, Texas, United States of America; UNILAB Research Center for Chemical Reaction Engineering, CHINA

## Abstract

Human networks and engineered systems are traditionally designed to maximize efficiency. Ecosystems on the other hand, achieve long-term robustness and sustainability by maintaining a unique balance between pathway efficiency and redundancy, measured in terms of the number of flow pathways available for a given unit of flow at any node in the network. Translating this flow-based ecosystem robustness into an engineering context supports the creation of new robust and sustainable design guidelines for engineered systems. Thermodynamic cycles provide good examples of human systems where simple and clearly defined modifications can be made to increase efficiency. Twenty-three variations on the Brayton and Rankine cycles are used to understand the relationship between design decisions that maximize a system’s efficient use of energy (measured by thermodynamic first law efficiency) and ecological measures of robustness and structural efficiency. The results reveal that thermodynamic efficiency and ecological pathway efficiency do not always correlate and that while on average modifications to increase energy efficiency reduce the robustness of the system, the engineering understanding of ecological network design presented here can enable decisions that are able to increase both energy efficiency *and* robustness.

## Introduction

### Sustainable systems

Sustainability of a system can be defined as its ability to maintain function in the present and future, despite fluctuations in inputs, demand, and the surroundings [1]. Many considerations go into defining sustainability of systems, including their social, economic, and environmental aspects [2–4]. Robustness is an important aspect of sustainability in systems as it can enhance the ability of a system to function effectively under different kinds of disturbances [3, 5]. Designing for robustness is a common goal across many disciplines that deal with systems, for example design theory and methodology. Metrics like Taguchi’s signal to noise ratio offer design guidance for dealing with unwanted input variations [6–8], the introduction of the signal to noise ratio advanced the way performance was balanced with qualities such as robustness in system designs. Over the years fields such as quality engineering and statistics have spurred variations to Taguchi’s methods, as well as other approaches that have lead to improvements in the robustness of systems and products [7, 9, 10]. These approaches are based on optimizing the effect of operational parameters on the performance measures of a system. Currently, there are no standard system level measures of robustness and sustainability for human networks that take into account both the topology and flow organization. Robustness for ecosystems on the other hand is well defined with a detailed quantitative formulation that considers both those attributes.

Successful ecosystems are those that are both efficient and robust [11], with flow path redundancy that enables them to deal with disturbances [12]. Efficiency and redundancy in ecological network pathways have been extensively investigated in the past two to three decades [12, 13]. Ecological network analysis (ENA) is a technique that allows for quantification of characteristics such as efficiency and redundancy, utilizing metrics to enable comparisons between different systems or system states. These metrics are calculated using information about the network structure (the presence or absence of a directed connection between two species) and network flow information (the magnitude of material/energy in those connections).

### Ecological network analysis for human networks

The application of ecosystem metrics, coupled with the characteristic ecosystem values, to human systems may provide unique design insights into mimicking the sustainability of nature. Biological ecosystems and industrial resource networks (networks of interacting industries) both represent a collection of entities (species and industries respectively) that exchange material and energy. Analogies between these two network types have been studied at a basic structural level [14–17]. The analogy’s application is based on the hypothesis that when industrial systems mimic features of ecosystems they are able to approach the sustainable and efficient functional characteristics of biological ecosystems. Ecological flow-based metrics have been applied to a few human networks. Economic networks have been investigated in terms of the connection between economic growth and some ENA metrics, clearly defining them from an economic standpoint [18–21]. Three Italian water networks were modified to reduce leakages in the system and the resultant change in ENA metrics was noted in [22]. Power grids have also been studied specifically using the ENA metric robustness for network redesign [23–25]. This ENA metric robustness is of particular interest for designers as it offers a straightforward way of quantifying a system’s robustness to perturbations, a major factor in determining a system’s sustainability. These studies however still leave questions as to what the ecosystem metrics, and specifically ecosystem robustness, mean from a general energy efficiency standpoint. Answering this question will enable a broader application of ENA metrics and ecosystem characteristics to engineering and energy systems design. Engineers are very familiar with energy efficiency and thermodynamic power cycles. Hence, connecting ecological robustness, and the balance between pathway efficiency and redundancy that define ecological robustness, with energy efficiency may provide designers and decision makers with a fundamental thermodynamics based understanding of ENA metrics. Panyam *et al*. [26] addressed this from a high level first-law efficiency standpoint for Rankine and Brayton cycles. Recently, the ecological metric ascendency has also been connected to exergy efficiencies of Rankine cycles [27]. The analysis by Panyam et al. [26] is extended here to deepen the energy-based understanding of ENA metrics and ecosystem performance.

### Thermodynamic principles and sustainability

Thermodynamic efficiency represents the efficiency with which the total available energy is used [28]. This definition relates thermodynamic efficiency to sustainability and as a result there have been many studies connecting thermodynamic principles with sustainability [2, 5, 29, 30]. Most of these works however, focus on *qualitative* analyses, with an exception of [30], which quantitatively investigated the ENA structural metric cyclicity using thermodynamic power cycles. The work correlates changes in thermodynamic efficiency to changes in the ecological metric cyclicity for a group of thermodynamic systems. Their results suggested that increases in thermal efficiency, indicating efficiency improvements in the use of input energy, correlated with higher cyclicity values, a characteristic of biological ecosystems. Cyclicity increases can thus be understood to quantify improvements in how a system uses the materials and energy available. The bio-inspired design of human networks for sustainability has since that work been further developed to use more complex flow-based metrics from ENA. As a result, there is a need for these flow-based metrics, including robustness, to be investigated and understood in a similar manner.

Similar to how ecosystems see an increase in pathway efficiency through maturation, efficiency of thermodynamic cycles can be increased through physical modifications. This provides an opportunity to test the results of well understood network modifications for energy efficiency on the resultant response of ecosystem metrics. Two basic thermodynamic systems are used here: Rankine and Brayton power cycles. These cycles are used due to their basic network structure and as they are both commonly used engineering systems with clear definition of efficiency. Their is also a precedent for their use to help understand engineering meaning in ecosystem characteristics in a past publication [30]. Brayton and Rankine cycles produce power by using energy in the working fluid, water and air respectively, via thermodynamic processes. These transformation and exchange processes are analogous to the prey-predator exchanges in ecosystems. First law efficiency (*η*_*I*_) measures the net energy output in terms of the total input energy for a thermodynamic system. The relationships between thermodynamic efficiency and four ecological flow metrics, robustness (*R*), *ASC/DC*, *AMI* and *H*, are processed for twenty three Rankine and Brayton cycles (one set of variations was done on the Brayton cycle and two are done for the Rankine cycle). The variations made to the cycles are of increasing complexity and are all done with the goal of increasing *η*_*I*_. The process is set up to result in the creation of an thermodynamics-based understanding of these ecosystem measures.

## Methods

### Thermodynamic power cycles

**Fig 1:**
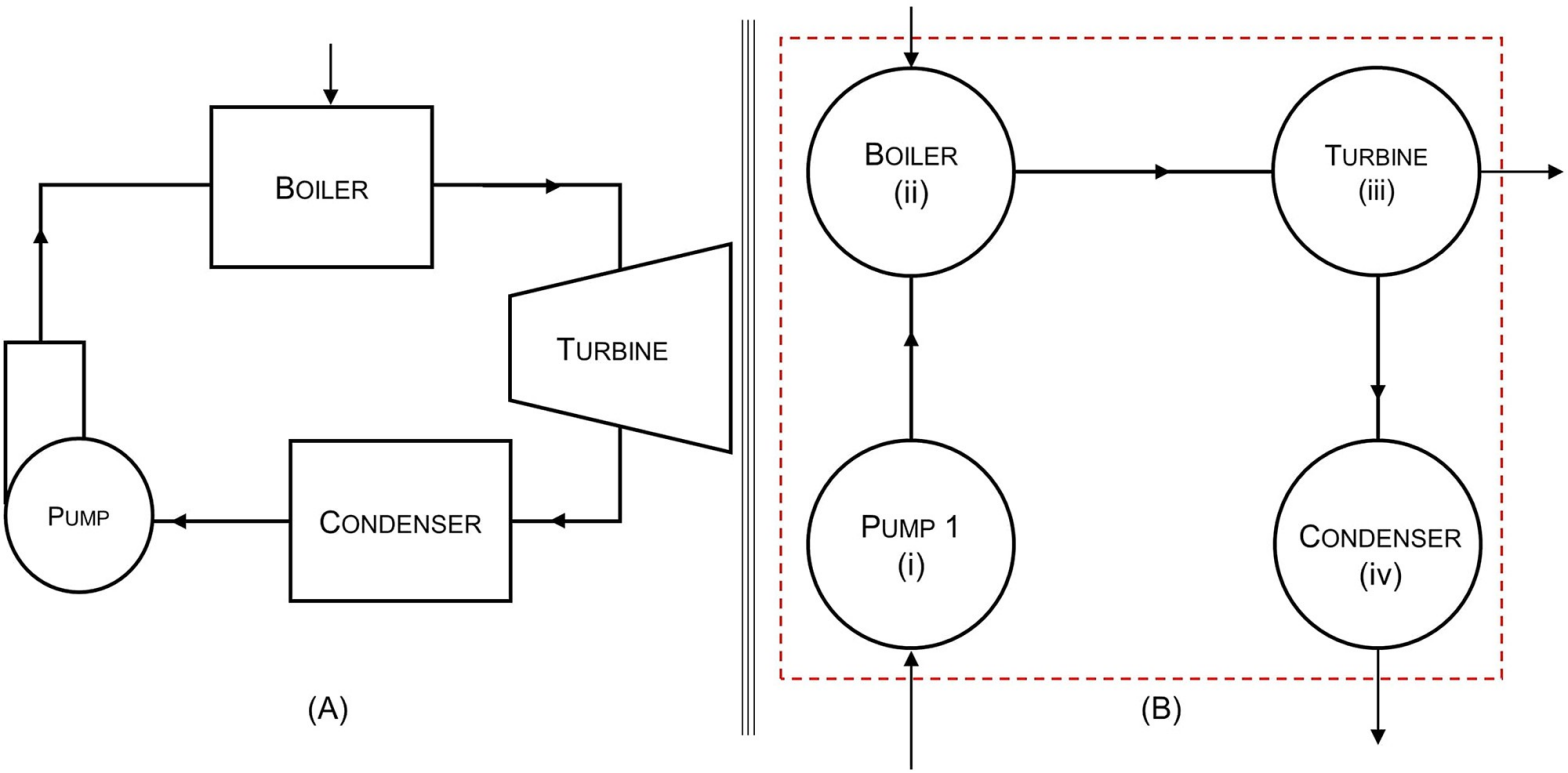
(A) An idealized equipment diagram and (B) A node diagram for the simple Rankine cycle. The red dotted square indicates the system boundary.

Seven modifications of the ideal Brayton cycle and fourteen modifications of the ideal Rankine cycle were used in the analysis. These types of cycles are widely used in jet engines (Brayton cycle) and power plants (Rankine cycle). A basic Brayton cycle consists of three components: compressor, combustor and turbine. The compressor draws in air from the atmosphere and compresses it, resulting in an increase in temperature and pressure. The high temperature air is mixed with fuel for combustion and the resulting high temperature gases propel the turbine. Part of the work output of the turbine is used to power the compressor creating a connection between the two components. A basic Rankine cycle consists of four components: pump, boiler, turbine, and condenser as shown in Fig 1. Water passes through the different components, converting from a liquid state to a vapor state and back. Heat and work are supplied by external sources as required by the boiler and pump respectively. The turbine outputs useful work and any leftover energy in the working fluid is exported in the form of heat by the condenser. Inputs to and outputs from the system are denoted by arrows crossing the boundary (dashed line) in the node diagram of Fig 1. The addition of components such as feed-water heaters (FWHs), reheaters, and intercoolers increases the thermodynamic efficiency of the Rankine and Brayton cycles [31]. These components harness the energy left over in the exhaust gases, usually in the form of waste heat, sending it back to earlier stages in the system where heat is required. These modifications decrease the amount of total heat energy required for system functioning. The source and sink temperatures of each cycle are kept constant across all the variations investigated here to ensure accurate comparisons, Table 1.

**Table 1:** Initial state point data used for the ideal Rankine and Brayton cycles.

Rankine cycles—Water	Brayton cycles—Air
*T*_*min*_ = 318.9 K	*T*_*min*_ = 288.2 K
*T*_*max*_ = 873.2 K	*T*_*max*_ = 1273 K
*P*_*pump*1,*input*_ = 10 kPa	*P*_*compressor,input*_ = 100 kPa
*P*_*boiler,input*_ = 15000 kPa	*r*_*p*_ = 10 (pressure ratio)
*η*_*C*_ = 0.634	*η*_*C*_ = 0.773

The energy exchanges between any two components in the cycle can be calculated using the enthalpy of the working fluid entering and exiting the components. Energy in the form of heat and work input to and output from the system are calculated from Eqs 1–3. The enthalpies, work, and heat for the cycles were calculated using Engineering Equation Solver (EES) version V8.881-3D. State point data for the basic Rankine cycle of Fig 1 is given in Table 2. Thermal efficiency calculated using Eq 4 uses the information in Table 2. Thermal efficiency is the ratio of total useful output from a cycle to the total energy input (Eq 5). The Carnot efficiency, given by Eq 4 specifies the maximum possible thermal efficiency that can be achieved by a power cycle operating between a given source and sink temperature [31].

$$W_{in,i}=(h_{exit}-h_{inlet}{)}_{compressor,pump}$$ (1)

$$W_{out,i}=(h_{exit}-h_{inlet}{)}_{turbine}$$ (2)

$$Q_{in,i}=(h_{exit}-h_{inlet}{)}_{boiler,combustor}$$ (3)

$$\begin{array}{c} \eta_{C}=1-\frac{T_{min}}{T_{max}} \end{array}$$ (4)

$$\begin{array}{c} \eta_{I}=\frac{\sum_{i}(W_{out,i}+W_{in,i})}{\sum_{i}(Q_{in,i})} \end{array}$$ (5)

**Table 2:** Energy information (kJ/kg) used to fill in the flow matrix [T] for the simple Rankine cycle of Fig 1, calculated using the temperature and pressure information in Table 1.

	T (K)	h (kJ/kg)
into (i)	318.9 (Saturated liquid)	191.8
out of (i) and into (ii)	319.5	206.9
out of (ii) and into (iii)	873.2	3582
out of (iii) and into (iv)	318.9 (Saturated vapor)	2114
out of (iv) and into environment	318.9 (Saturated liquid)	191.8

### Ecological flow-based analyses

Organization of energy/material flows in ecosystems can be analyzed using an ecological network analysis (ENA). The flow matrix, denoted by [**T**], is the basis for an ENA flow analysis. [**T**] is a (*N*+3) x (*N*+3) matrix that contains quantitative flow magnitudes for all of the internal interactions from node *i* to node *j*, inputs, outputs and dissipation for an ecosystem network [11]. The number of actors in the network is contained in *N*. The three additional rows and columns beyond *N* represent the inputs, outputs and dissipation at each node that cross the system boundary.

**Fig 2:**
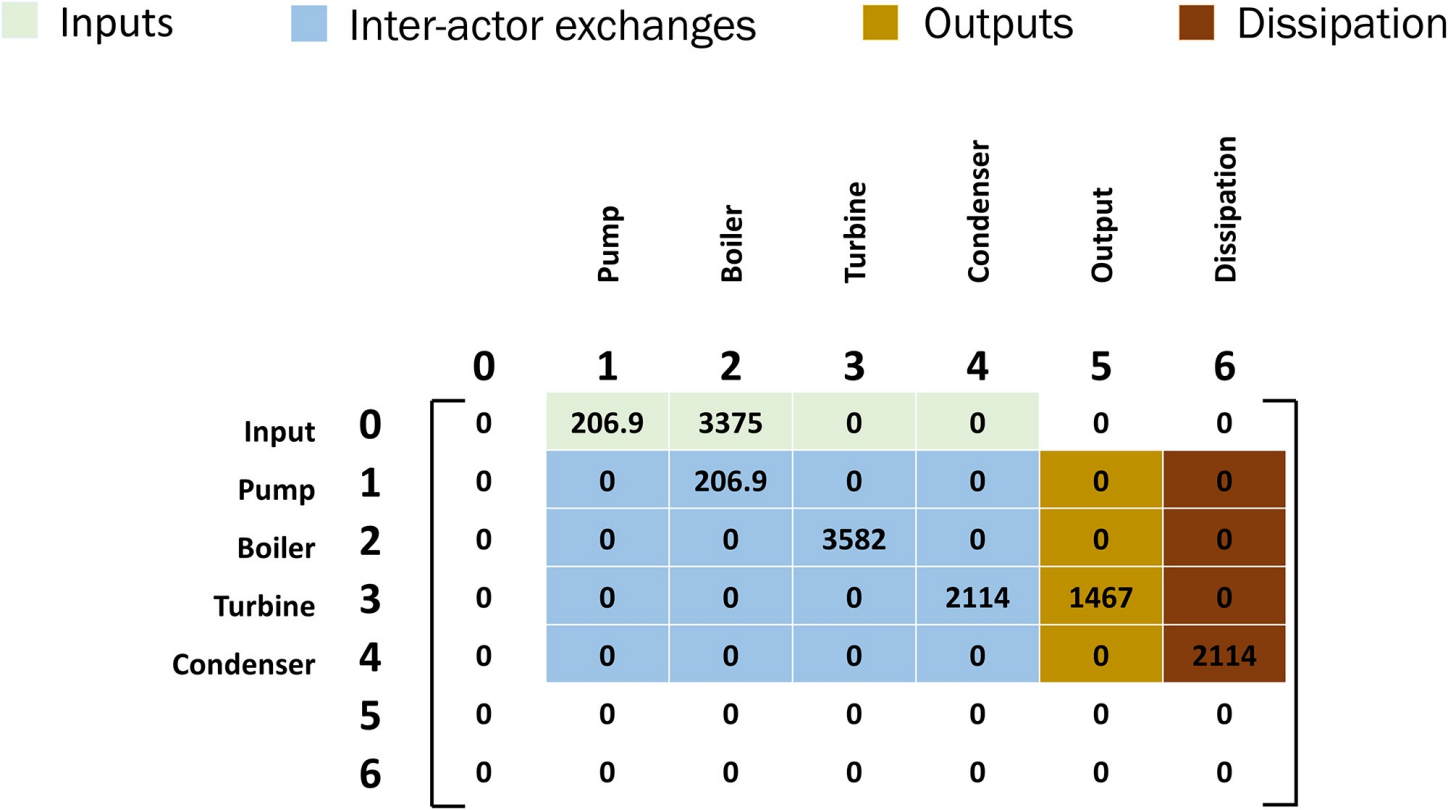
Flow matrix [T] for the simple Rankine cycle of Fig 1. All the flows are measured in [kJ/kg] of energy. Flow is documented as going from rows to (i) to columns (j). Row 0 is inputs to the system, column 5 contains outputs from the system and column 6 is the dissipation to the outside environment.

The ENA of power cycles here considers each component in the thermodynamic cycles an actor. Fig 2 shows a flow matrix for the basic Rankine cycle of Fig 1. Energy values (kJ/kg) are from the enthalpy, heat, and work information in Table 2. S1–S5 Figs provide additional illustrations of flow diagrams and flow matrices for the first cycle in each modified series of the Brayton and Rankine cycles.

Total system throughput (*TSTp*, Eq 6) is the sum of *all* the flows interacting with the system. The ecologist Ulanowicz [11] developed a metric to quantify the robustness of ecosystems as a function of two opposing measures: pathway efficiency and redundancy [11, 12]. Pathway efficiency and redundancy are measured by the ENA metrics ascendency (*ASC*) and development capacity (*DC*) (given by Eqs 8 and 10). A higher *DC* can signify that on average two points in the system will experience a higher redundancy in the pathways connecting them. *ASC* increases and *DC* tends to drop as the system becomes more constrained and organized [26]. Average mutual information (*AMI*) and Shannon index (*H*) are the dimensionless versions of *ASC* and *DC*, respectively (obtained by normalizing with *TSTp*). Total system overhead (*TSO*) is the difference between *ASC* and *DC* and represents the part of the network that can still be converted to increasing pathway efficiency. ENA metrics, these included, are derived from information theory. The ratio *ASC/DC* (or *AMI/H*) represents the degree of system order on a scale of zero to one [23], presenting a dimensionless measure of pathway efficiency. Pathway efficiency, referred to as simply “efficiency” in ecological literature, should be noted to be different from engineering efficiency, as will be discussed later.

$$\begin{array}{c} TSTp=\sum_{i=0}^{N+2}\sum_{j=0}^{N+2}T_{ij} \end{array}$$ (6)

$$\begin{array}{c} AMI=-\sum_{i=0}^{N+2}\sum_{j=0}^{N+2}((\frac{T_{ij}}{TSTp}\times {\text{log}}_{2}(\frac{T_{ij}\times TSTp}{\sum_{m=0}^{N+2}T_{im}\times \sum_{n=0}^{N+2}T_{nj}}))) \end{array}$$ (7)

$$\begin{array}{c} ASC=TSTp\times AMI \end{array}$$ (8)

$$\begin{array}{c} H=-\sum_{i=0}^{N+2}\sum_{j=0}^{N+2}log_{2}(\frac{T_{ij}}{TSTp}) \end{array}$$ (9)

$$\begin{array}{c} DC=TSTp\times H \end{array}$$ (10)

$$\begin{array}{c} R=-(\frac{ASC}{DC})\times ln(\frac{ASC}{DC}) \end{array}$$ (11)

Natural systems have been found by ecologists as having up to 25% redundancy [22]. This redundancy is believed to equip the system with additional flow paths and allows for effective reorganization of the network in the face of disturbances. Redundancy in human networks and systems, however, is usually seen as an inefficiency that increases costs. Redundancy in these systems/networks is usually traded for efficiency and profit maximization by optimizing the network configuration.

**Fig 3:**
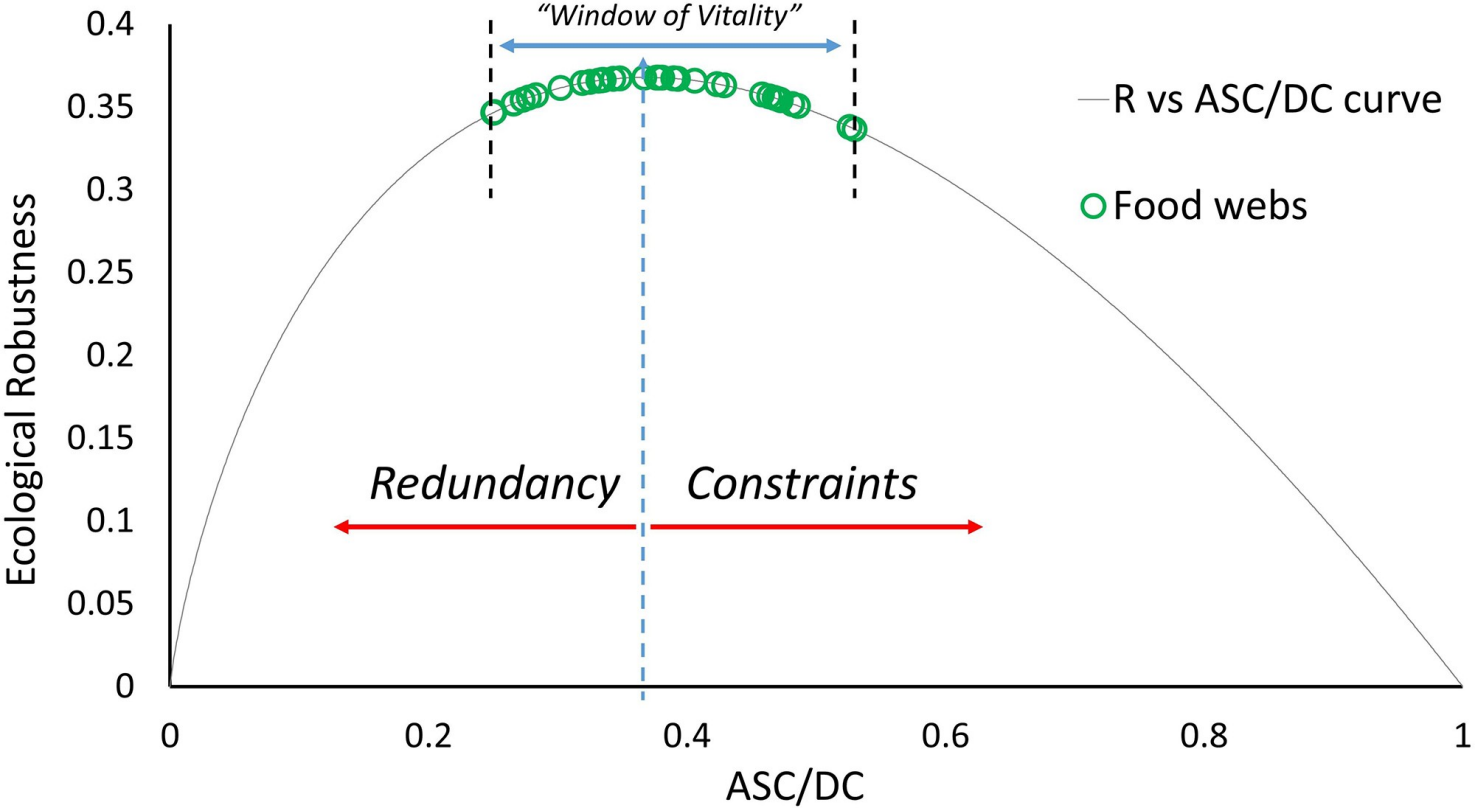
Ecological robustness plotted against the ratio *ASC/DC*. Thirty eight food webs taken from the datasets of [32] are plotted on the curve fit, illustrating the “window of vitality” that most ecosystems reside in. The peak of the curve corresponds to a *R* value of 0.368 and an *ASC/DC* value of 0.368. Figure used with permission from [26].

Ecological robustness (Eq 11) is the product of the degree of system order (*ASC/DC*) and the negative of its natural logarithm (*ln*(*ASC*/*DC*)). The formulation of ecological robustness (*R*) weights redundancy slightly more than efficiency, placing the peak robustness value at *ASC*/*DC* = 0.368. Robustness is zero when the ratio *ASC/DC* is zero and one. The “window of vitality” [18, 33], shown in Fig 3 describes the peak when *R* is plotted against *ASC/DC* where most ecosystems can be found [32]. Ecologists hypothesize that the network configuration corresponding to the peak of the curve is indicative of Nature having achieved a successful balance between pathway efficiency and redundancy, that results in a robust and sustainable state [3, 11]. Human networks often experience disturbances analogous to ecosystems: for example, weather changes and variations in supply and demand. The ecological robustness metric is thus potentially very useful to quantify the capacity of human networks—in terms of their organization—to sustain functioning during disruptions.

## Results: Ecological flow based analysis of thermodynamic power cycles

Thermal efficiencies and four flow-based ecosystem metrics: Shannon Index (*H*, Eq 9), average mutual information (*AMI*, Eq 7), *ASC/DC*, and ecological robustness (*R*, Eq 11) were evaluated for 23 Brayton and Rankine power cycles. The results are listed in Table 3. The modifications on the basic Brayton cycle include the addition of regeneration, intercooling and reheat, creating a total of 8 different Brayton cycles with increasing efficiencies. B represents the basic Brayton cycle and B1 has added regeneration B2 has added intercooling and reheat (resulting in 2 turbines), and B3 is the same as B2 but with 3 turbines. Modifications to the Rankine cycle were similar but were carried out as two sets of variations, creating a total of 15 Rankine cycles: 1) adding an increasing number of open feedwater heaters to the simple Rankine cycle (RO1 adds one open feedwater heater and RO2 adds two) and 2) adding reheat and open feedwater heaters to the simple Rankine cycle (RRO1 adds reheat and one open feedwater heater, RRO2 adds reheat and two open feedwater heaters, etc.). Average values of ecological robustness and *ASC/DC* for 38 food webs from [32] are also listed for comparison in Table 3.

**Table 3:** The sets of modifications made to each cycle and the corresponding changes in thermal efficiency (*η*_*I*_), average mutual information (*AMI*), the Shannon Index (*H*), the degree of system order (*ASC/DC*), and ecosystem robustness (*R*). B1-7: Gradual addition of regeneration, intercooling and reheat. RO1-8: Gradual addition of open feed-water heaters. RRO1-6: Gradual addition of reheat and open feed-water heaters.

Cycle	*η*_*I*_	AMI	H	ASC/DC	R
**FWs average**	-	1.610	3.880	0.414	0.365
**B**	0.482	1.495	2.588	0.578	0.317
**B1**	0.579	1.676	2.999	0.559	0.325
**B2**	0.685	2.441	3.711	0.658	0.276
**B3**	0.718	2.957	4.133	0.715	0.240
**B4**	0.733	3.342	4.450	0.751	0.215
**B5**	0.742	3.635	4.718	0.770	0.201
**B6**	0.748	3.886	4.936	0.787	0.188
**B7**	0.751	4.106	5.120	0.802	0.177
**R**	0.430	1.968	2.410	0.817	0.166
**RO1**	0.453	2.164	2.880	0.751	0.251
**RO2**	0.472	2.367	3.202	0.739	0.223
**RO3**	0.476	2.574	3.392	0.759	0.209
**RO4**	0.479	2.742	3.617	0.758	0.210
**RO5**	0.480	2.929	3.792	0.772	0.200
**RO6**	0.482	3.032	3.898	0.778	0.196
**RO7**	0.482	3.195	4.215	0.758	0.210
**RO8**	0.483	3.414	4.226	0.802	0.172
**RRO1**	0.470	2.007	3.347	0.600	0.301
**RRO2**	0.483	2.082	3.558	0.585	0.314
**RRO3**	0.488	2.288	3.673	0.623	0.295
**RRO4**	0.491	2.444	3.811	0.641	0.285
**RRO5**	0.492	2.608	3.949	0.661	0.274
**RRO6**	0.493	2.691	4.024	0.669	0.269

**Fig 4:**
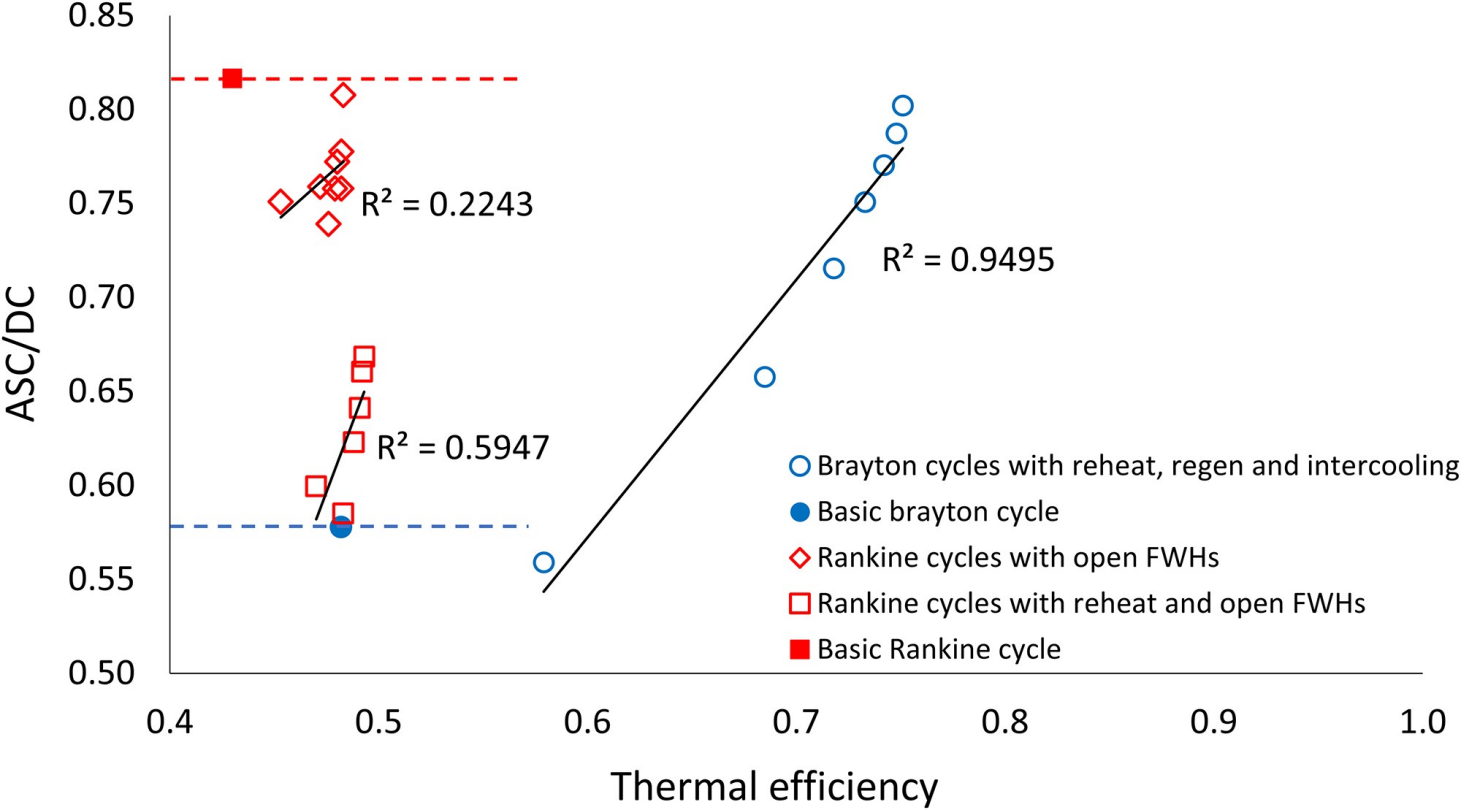
Thermal efficiency plotted against the ratio *ASC/DC* for the three sets of thermodynamic cycle improvements. The *ASC/DC* values of the basic Brayton and Rankine cycles are highlighted by the red and blue dotted lines.

**Fig 5:**
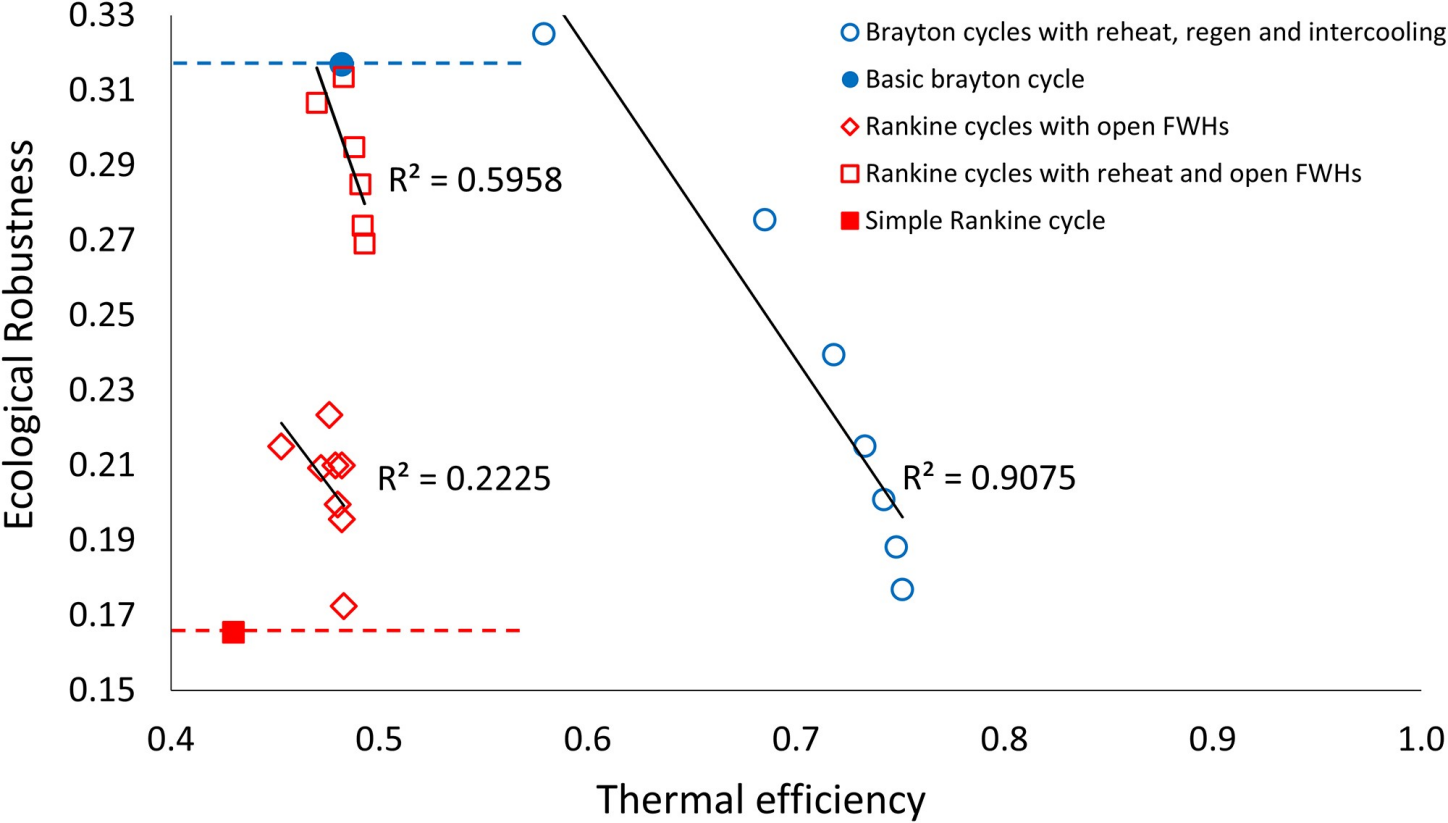
Thermal efficiency plotted against ecological robustness for the three sets of thermodynamic cycle improvements. The *R* values of the basic Brayton and Rankine cycles are highlighted by the red and blue dotted lines.

**Fig 6:**
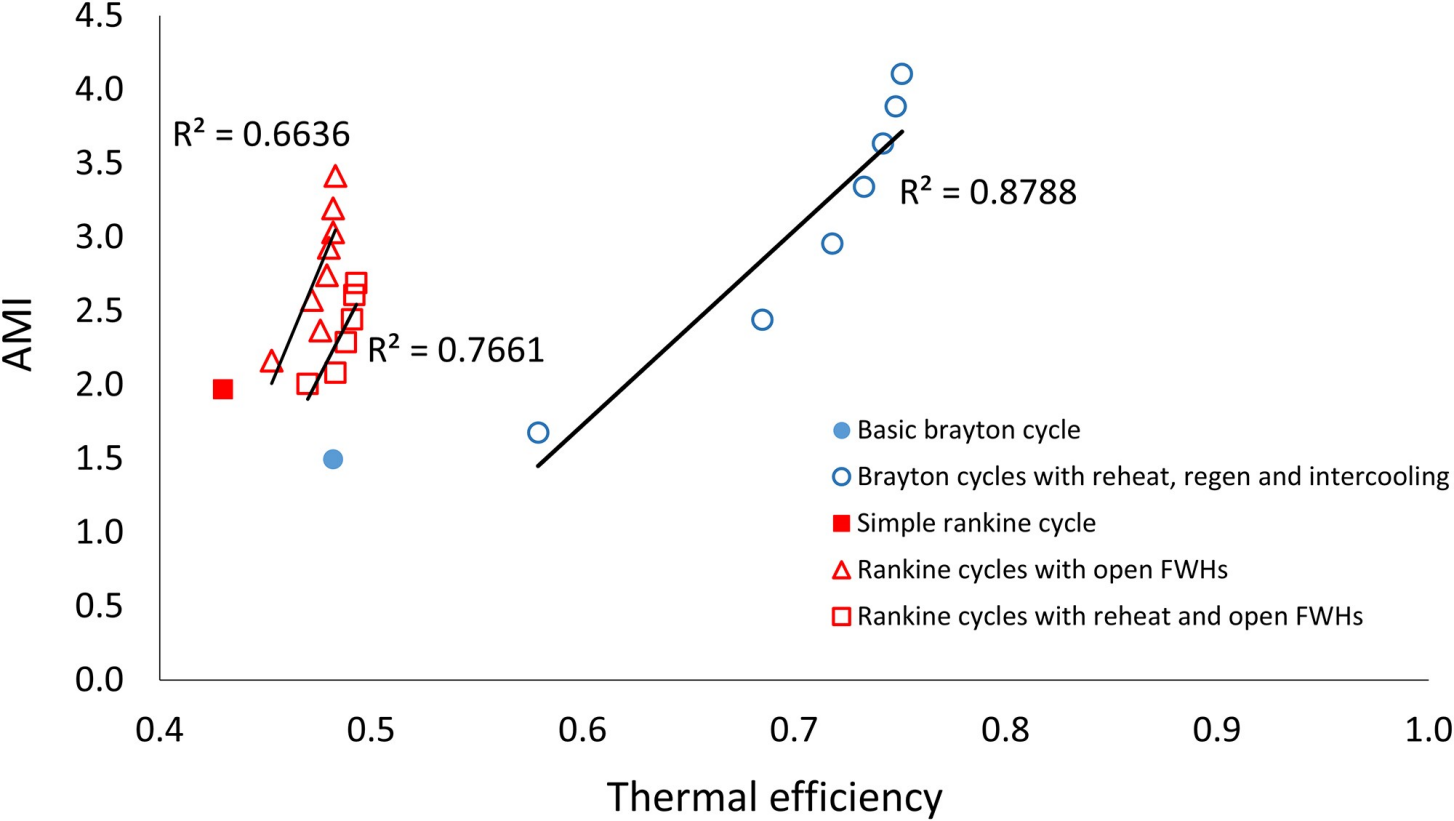
Thermal efficiency plotted against average mutual information (*AMI*) for the three sets of thermodynamic cycle improvements.

**Fig 7:**
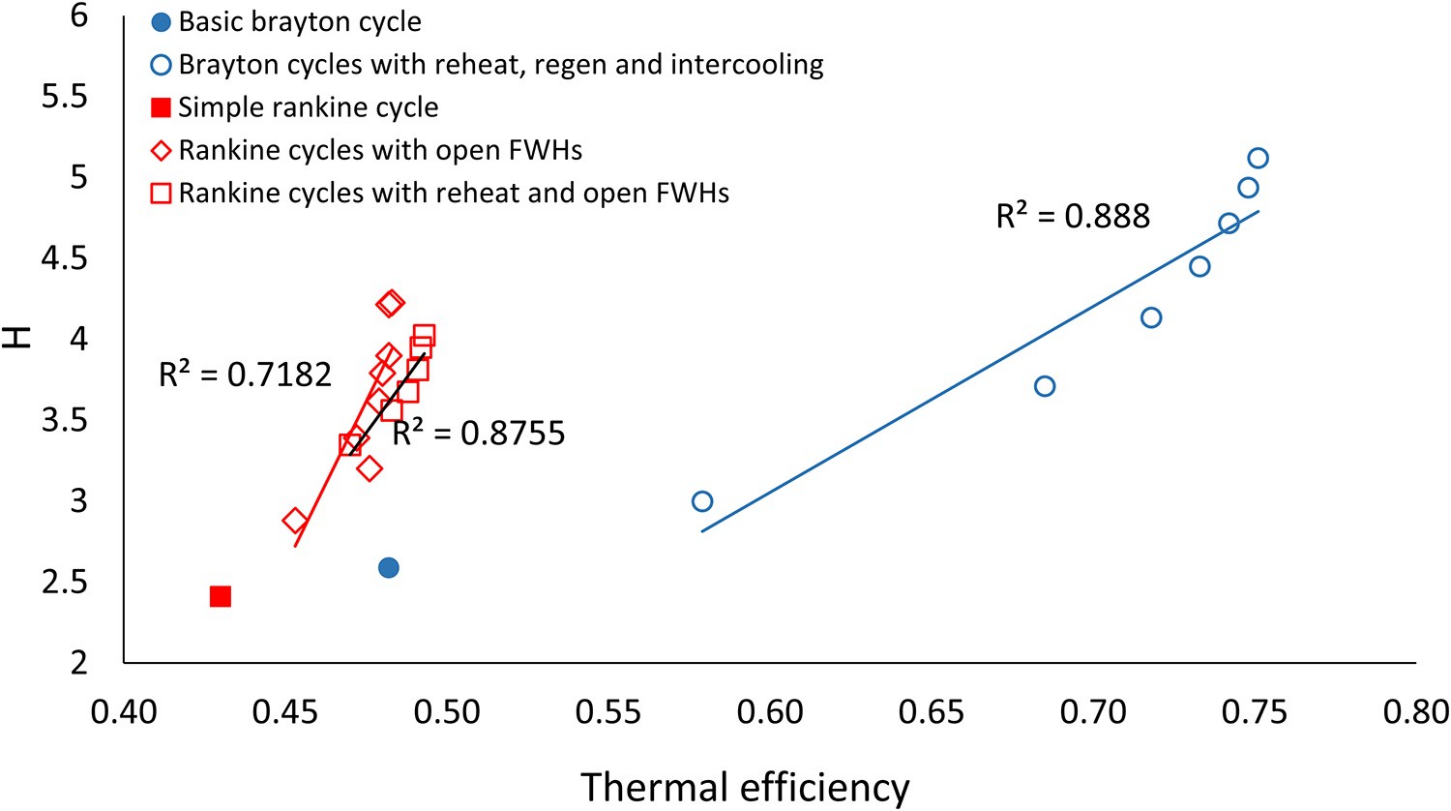
Thermal efficiency plotted against Shannon Index (*H*) for the three sets of thermodynamic cycle improvements.

Fig 4 shows how the ratio ascendency to development capacity (*ASC/DC*) for the thermodynamic cycles varies with increasing thermal efficiency. Fig 5 shows ecological robustness (Fig 5) for the thermodynamic power cycles with respect to increasing thermal efficiency. Both Figs 4 and 5 show the corresponding linear trend lines. Generally increasing and decreasing trends are seen for *ASC/DC* and *R*, respectively, with increasing efficiencies, with some exceptions. Fig 6 shows the relationship between thermal efficiency and *AMI*. Fig 7 depicts the relationship between thermal efficiency and Shannon Index (*H*). Strictly increasing trends are observed between the ENA metrics and thermal efficiency for these two figures.

## Discussion

Intended and unintended modifications can both cause system development and evolution. The ability to design human systems results in most connections being very intentional. Unintended linkages can emerge in some systems however, like trade and economic networks, due to extrinsic constraints imposed on the system (e.g. profit maximization and regulations). Most ecosystems evolve due to such externally imposed requirements. The different variations of Rankine and Brayton cycles analyzed here consist of intended modifications to increase the thermal efficiency. A benefit to the analysis presented here is that these purposeful design changes have clear effects. Although network robustness is not usually considered in the design of these cycles, they provide an opportunity to compare network modifications made to increase work output with the evolution seen in naturally sustainable ecosystems. This would lead to an understanding as to whether maximizing output aids or impedes the development of sustainable human systems through bio-mimicry.

The discussion focuses on two interesting findings: 1) the relationship of thermal efficiency with ecological pathway efficiency (*ASC/DC*) and robustness and 2) the correlations of thermal efficiency with *AMI* and *H*.

### *ASC/DC* and 1^st^ law efficiency

Efficiency from a thermodynamic perspective measures the useful output generated from a specified input. Ecological network efficiency however, measures the efficiency of the network structure in terms of the number of pathways available for any given unit of flow. The design goals of engineered systems are often seen as striving towards a highly organized structure, and as a result one would expect higher *ASC/DC* values. This is not necessarily the case though and is most likely the result of some human systems containing critical components requiring redundant supply pathways that function as backup in cases of system malfunctions. An *ASC/DC* value closer to one indicates a more streamlined network structure with less pathway diversity. Values of *ASC/DC* closer to zero indicate more pathway diversity, or a higher number of pathways available for any given unit of flow. *ASC/DC* values for some human water networks [34] were found to have values further from one, ranging from 0.48–0.61, which are closer to those found in Nature. A Chesapeake Bay ecosystem was found to have an *ASC/DC* of 0.60 [35] and a South Florida Everglades ecosystem had an *ASC/DC* of 0.55 [36].

The set of 38 food webs used here have an average *ASC/DC* value of 0.414 (first row of Table 3). All of the thermodynamic cycles investigated here in contrast have higher values of *ASC/DC*, ranging from 0.57 to 0.81. Despite the conceptual difference between engineering efficiency and ecosystem efficiency, the results comparing *ASC/DC* to *η*_*I*_ in Fig 4 show that for many of the cycles with a high thermal efficiency this also equates to a high pathway efficiency. This comparison suggests that design decisions made to increase *η*_*I*_ in thermodynamic power systems mimic a minimization of pathway diversity, resulting in a system that is significantly more streamlined in structure than what is found on average in ecosystems. This relationship, between engineering efficiency increases and ecological efficiency increases, however is not found to be strictly positive within each set of modifications. The relationship also differs between the two different types of power cycles.

The B1 to B7 Brayton cycles show an increasing trend between *ASC*/*DC* and thermal efficiency, as seen in Fig 4. The Brayton cycle B1 has the lowest *ASC*/*DC* and B7 has the highest, 0.56 and 0.80 respectively, resulting in a high *R*^2^ value of 0.95 that suggests a strong connection between increases in thermal efficiency and increases in ecological pathway efficiency. The two sets of Rankine cycle variations, with open feedwater heaters and with reheat *and* open feedwater heaters, both show an initial negative relation between *ASC*/*DC* and thermal efficiency from the first modification to the second resulting in much lower *R*^2^ values of 0.22 and 0.59 respectively. This corresponds conceptually to the new added components requiring multiple additional pathways as a pattern is set up that increase the ratio between pathways and components. After this first modification though the relationship is positive. This inconsistent trend in the Rankine cycle modifications, one that is not seen for the Brayton cycles, could be due to the relatively small increases in thermal efficiency between the different Rankine cycles. The *η*_*I*_ increases are small when compared to the increases made between the different Brayton cycles. Fig 4 highlights the small changes in *η*_*I*_ for the Rankine cycles corresponding to larger changes in *ASC*/*DC* values. Amongst the two type of modifications made to the Rankine cycles Fig 4 also shows that while both modifications increase the pathway redundancy (which results in changes to ecological robustness as discussed in the next subsection), adding reheat *and* open feed water heaters introduces more redundancy in flow paths than adding only open feed water heaters. The correlations in Fig 4 indicate that improving the energy efficiency of human systems does *not always* result in more streamlined flow paths, but can sometimes lead to redundant flow paths. The responses of the two sets of power cycles show that while that ecological pathway efficiency does not perfectly correlate with engineering/thermal efficiency the two generally increase with each other amongst specific cycle modification sets.

### Ecological robustness and 1^st^ law efficiency

Systems with high *ASC/DC* values efficiently use a minimal number of pathways to deliver all the energy/materials needed to their species. These ecosystems however are vulnerable to disturbances: every pathway is crucial and if lost will result in some consumer need not being met. Systems with low *ASC/DC* values tend to be more resilient to disturbances, with redundant pathways to deliver energy and materials where they are needed. Extreme redundancy however can result in waste and increases in dissipation (longer pathways traveled), and higher costs in human engineered systems. Sustainable ecosystems have been found to cluster around what is known as the “window of vitality” (highlighted in Fig 3), that encompasses the maximum ecological robustness value of *R* = 0.367 or 1/*e*) [11, 12, 37]. That ecosystems tend to reside in this window is believed by ecologists to represent a unique balance achieved by nature between pathway efficiency and redundancy. All the thermodynamic cycles investigated here lie to the right of the window, suggesting that they have a lower robustness compared to ecosystems resulting from relatively high pathway efficiency (higher *ASC/DC* values). Fig 5 illustrates the generally negative correlation between *R* and *η*_*I*_ in each of the 3 sets of power cycle modifications (B2–7, RO1–8 and RRO1–6). Once again though this trend is not the rule, at least one variation in both the Rankine and Brayton cycles have higher ecological robustness *as well as* higher thermal efficiencies than their basic configurations. Fig 5 also highlights a significant difference in the ecological robustness values of the basic versions of the Rankine (*R* = 0.166) and the Brayton (*R* = 0.317) power cycles (red and blue dotted lines cutting the vertical axis).

**Fig 8:**
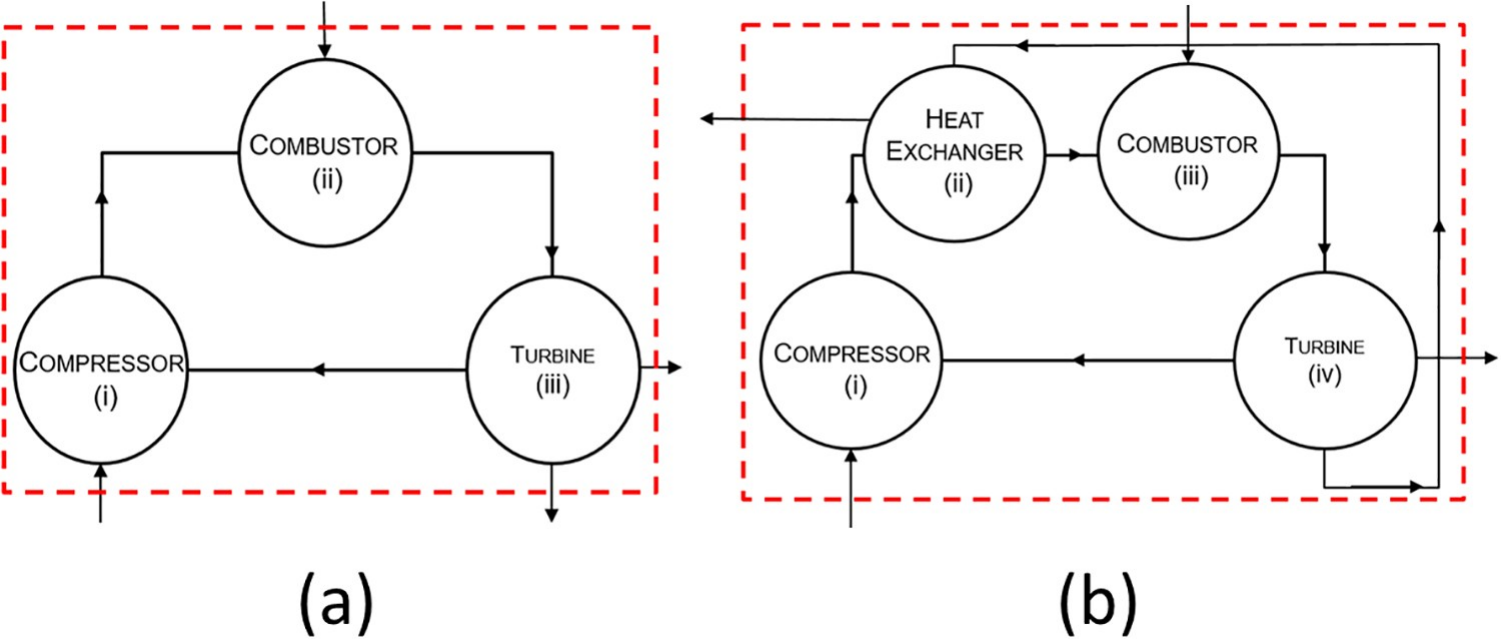
(a) Energy flow diagram for the basic Brayton cycle (B in Table 3) and (b) Energy flow diagram for the Brayton cycle with regeneration (B1 in Table 3). Energy flows are shown between components and across the system boundaries (denoted by dashed box).

Robustness increases immediately with the first modification for the Brayton cycles. This increase can be attributed to the structural changes that are meant to increase the thermal efficiency adding redundancy that increases the robustness. Adding regeneration to the basic Brayton cycle, (B) to (B1), both the thermal efficiency and ecological robustness increase (*η*_*I*_ = 0.48 to 0.58 and *R* = 0.32 to 0.33, see Table 3). This initial modification increasing both *η*_*I*_ and *R* is not seen for either of the two Rankine cycle modification sets and can be conceptually explained by looking at how the modification changes the structure of the cycle. The extra component added to the basic Brayton cycle, as seen in Fig 8, does not require *any* additional energy input (the new actor ii—heat exchanger, going from Fig 8a to 8b). All the other modifications of the Brayton cycles as well as all of the Rankine cycle variations (B2–7, RO1–8, and RRO1–6) conversely require *additional* energy to run the new components (pumps, compressors, and combustors). These subsequent modifications in both the Rankine and Brayton cycles increase the thermal efficiency but decrease both the pathway redundancy and the ecological robustness.

The trend seen in Fig 5, while generally negative, does show that it is possible to make design decisions that *both* increase engineering efficiency and robustness to disturbances. This discovery is especially important for systems that might have vulnerable customers or unstable weather for example. Rather than achieving energy efficiency through highly streamlined and pathway efficient organization, the analysis of ecological robustness on the power cycles has shown that systems that better take advantage of all available value inside their system boundaries may be able to achieve their energy efficiency goals without having to lose important redundancy. The results clearly show that the power cycles are able to increase their ecological robustness *and* their thermal efficiency at times, these +/+ scenarios upon investigation can be seen to be points where value (in the form of energy) remaining in the working fluid that originally was dumped to the surroundings is cleverly circulated back into the power cycle using components such as heat exchangers, intercoolers, reheaters and open feedwater pumps. The negative trend between thermal efficiency and ecological robustness, which is considered a measure of sustainability for ecosystems, also points out a possible incompatibility between the energy conservation perspective [5, 28, 29] and the network perspective of sustainability. A more holistic and possibly bio-inspired approach that considers both connectivity and energy related aspects may lead to system designs that are able to improve their overall sustainability without sacrificing engineering efficiency.

### AMI & Shannon Index and 1^st^ law efficiency

Correlating ecological metrics (*AMI* and Shannon Index) that go into defining ecosystem robustness with thermal efficiency revealed more consistent and stronger trends across variations for both Rankine and Brayton cycles.

Shannon Index (*H*) quantifies the total capacity for organization in an ecosystem. Average mutual information (*AMI*) quantifies the realized portion of that organizational capacity [11]. Ecosystems are hypothesized to increase both *AMI* and *H* as they evolve [38]. Thermodynamic power cycles also see an increase in *AMI* and *H* with modifications made to increase thermal efficiency. *AMI* and *H*, the basis for formulating both *R* and *ASC/DC*, were found here to have strong positive correlations with thermal efficiency (Figs 6 and 7). Unlike ecosystem robustness (*R*) and its dependent variable *ASC/DC*, the *AMI* and *H* for power cycles were found to strictly increase with increasing thermal efficiency (Figs 6 and 7).

The relationship between *AMI* and *H* resembles the relationship between thermal efficiency and Carnot efficiency. Carnot efficiency gives the maximum thermal efficiency that can be achieved by a power cycle operating between a given source and sink temperature. Thermodynamic first law efficiency is the *actual* realized efficiency. Irreversibility, the difference between maximum possible output and the actual output, arises due to entropy generation and prevents the thermal efficiency of these power cycles from ever reaching their Carnot efficiency. Similarly in ecosystems, pathway redundancy prevents *AMI* from ever reaching *H*. One difference however is that, for all the modified power cycles considered here, *H* changes but the Carnot efficiency remains the same as the basic version. This is because *H* depends on the structural potential of the system, which the Carnot efficiency is predetermined by the source and sink temperatures, which are kept constant across the power cycle variations.

The *R*^2^ values for the linear correlations between *AMI* and *η*_*I*_ (Fig 6) are 0.88, 0.66 and 0.76 for the modified B, RO and RRO series, respectively. The *R*^2^ values for the correlation between *H* and *η*_*I*_ (Fig 7) are 0.88, 0.72 and 0.87 for the modified B, RO and RRO series, respectively. Such a strong positive connection between quantitative measurements of ecosystem development and thermodynamic performance improvements suggests that the same fundamental principles may govern the development of both ecosystems and human systems. The authors remind the reader of the distinct difference between the pathway efficiency of ecosystems and the first law efficiency of thermodynamic power cycles(*η*_*I*_). The latter is with regard to maximizing the useful outputs obtained from system inputs (work out vs. heat in), while the former is related to the pathways between nodes—higher efficiency in this case involves fewer ways of traveling from point A to point B. If future analyses prove this true, it would result in two developments: 1) the study of ecosystems would become normative as opposed to the current descriptive efforts and 2) mimicking the development observed in naturally sustainable ecosystems in human systems also becomes normative as a result. The first addresses an issue that some ecologists have brought up regarding the existing discordant theories for the study of ecosystems [13, 29]. The second benefits the current efforts in the field of industrial ecology by providing norms for strengthening the analogy between ecological and human systems.

## Conclusions

The ecological flow metrics investigated here (*AMI*, *H*, *ASC/DC* and *R*) quantify pathway efficiency and robustness, describing the network characteristics and evolution of biological ecosystems. The strong correlations between thermal efficiency and the ecological measures *AMI* and *H* suggest that similar fundamental principles may govern the development of ecosystems and human systems. This has the potential to develop the study of natural ecosystems and industrial ecosystems into normative science. The flow metrics studied also suggest potential to provide inherently-sustainable tools for the design of human engineered systems. The application of these ecological metrics to thermodynamic power cycles provided a quantitative engineering-based understanding. Ecological pathway efficiency and thermal efficiency positively correlate among a single set of power cycle modifications, and although increases in thermal efficiency tend to reduce system robustness, the relationship is not a general one. The three types of power cycle modifications have suggested that the possibility of designing systems that have redundancy in their structure without sacrificing engineering efficiency may be realized using bio-inspiration, resulting in systems that improve both their robustness *and* their energy efficiency. Human systems, such as critical infrastructure networks, can experience economic instability, component failures, and climate disturbances. These threats suggest that human systems can benefit from mimicking ecosystem robustness levels, preventing efficiency focused design decisions that result in highly streamlined pathways causing the system to be vulnerable to disturbances. The continued application of ecosystem robustness to human systems will provide more insights into developing a holistic approach to assess sustainability of human systems that accounts for both the system form and function, energy conservation and system organization.

## Supporting information

S1 Fig Basic Brayton cycle. (a) Energy flow diagram, (b) Ecological flow matrix. Cp refers to compressor; Cb refers to combustor; T refers to turbine. The red dotted square indicates system boundary.
(TIF)

S2 Fig Brayton cycle with regeneration. (a) Energy flow diagram, (b) Ecological flow matrix. Cp refers to compressor; HX refers to regeneration heat exchanger; Cb refers to combustor; T refers to turbine. The red dotted square indicates system boundary.
(TIF)

S3 Fig Brayton cycle with regeneration, intercooling and reheat. (a) Energy flow diagram, (b) Ecological flow matrix. Cp refers to compressor; HX refers to regeneration heat exchanger; Cb refers to combustor; T refers to turbine. The red dotted square indicates system boundary.
(TIF)

S4 Fig Rankine cycle with 1 open feed water heater. (a) Energy flow diagram, (b) Ecological flow matrix. P refers to pump; OFWH refers to open feed water heater; B refers to boiler; T refers to turbine; C refers to condenser. The red dotted square indicates system boundary.
(TIF)

S5 Fig Rankine cycle with reheat and 1 open feed water heater. (a) Energy flow diagram, (b) Ecological flow matrix. P refers to pump; OFWH refers to open feed water heater; B refers to boiler; T refers to turbine; C refers to condenser. The red dotted square indicates system boundary.
(TIF)
