# Supplementary figures and images for: A quantitative engineering study of ecosystem robustness using thermodynamic power cycles as case studies
Source: PLoS One. 2019 Dec 31;14(12):e0226993. doi: 10.1371/journal.pone.0226993 (PMC6938333; doi:10.1371/journal.pone.0226993)

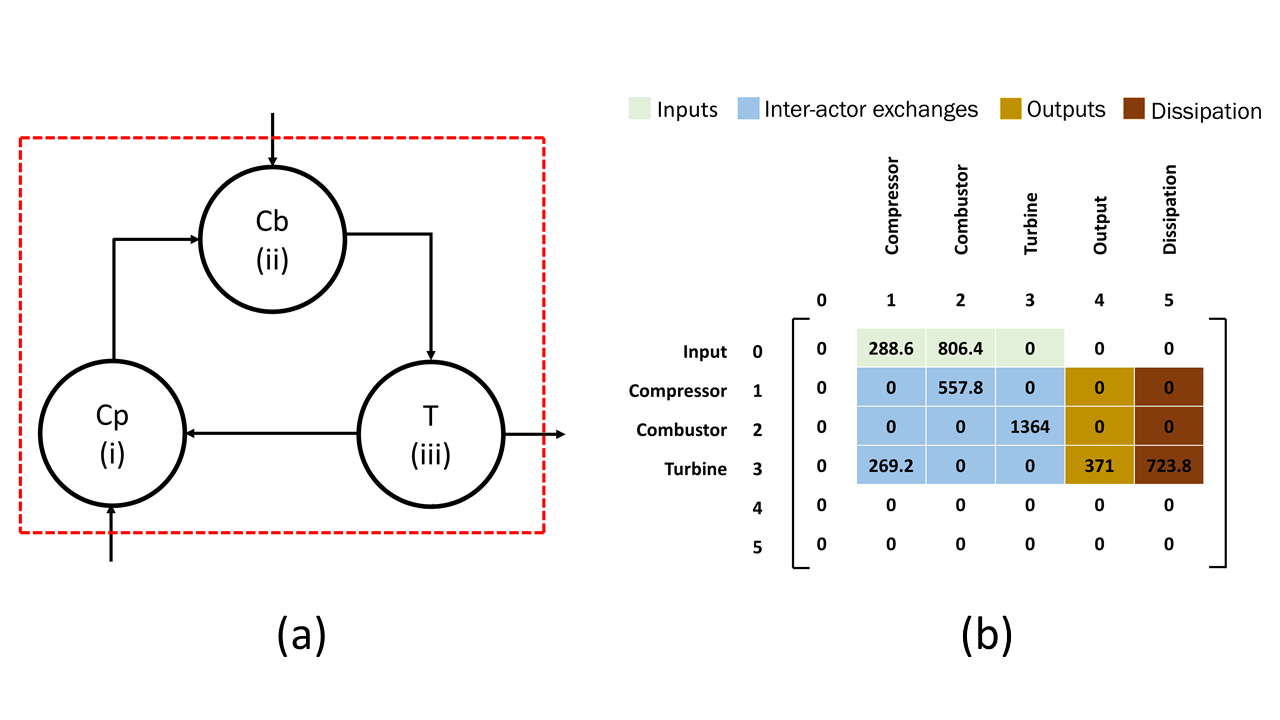

Supplement: S1 Fig — (a) Energy flow diagram, (b) Ecological flow matrix. Cp refers to compressor; Cb refers to combustor; T refers to turbine. The red dotted square indicates system boundary. (TIF) [file pone.0226993.s001.tif]

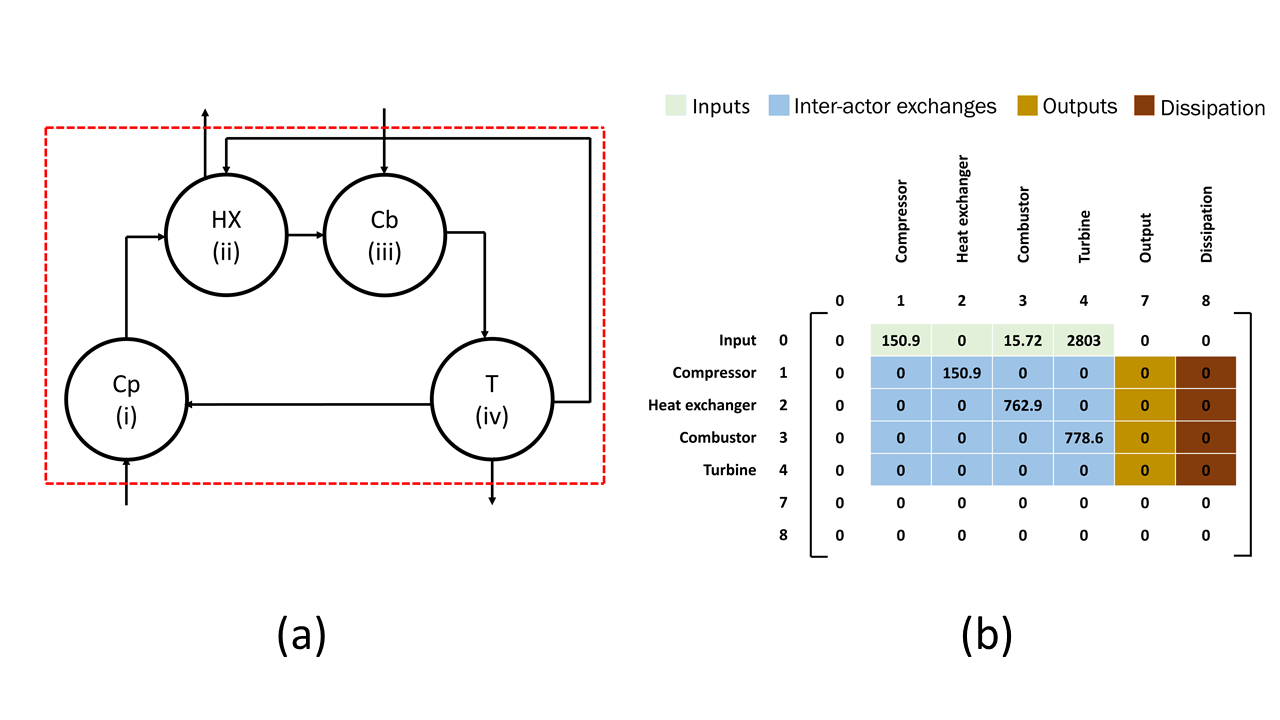

Supplement: S2 Fig — (a) Energy flow diagram, (b) Ecological flow matrix. Cp refers to compressor; HX refers to regeneration heat exchanger; Cb refers to combustor; T refers to turbine. The red dotted square indicates system boundary. (TIF) [file pone.0226993.s002.tif]

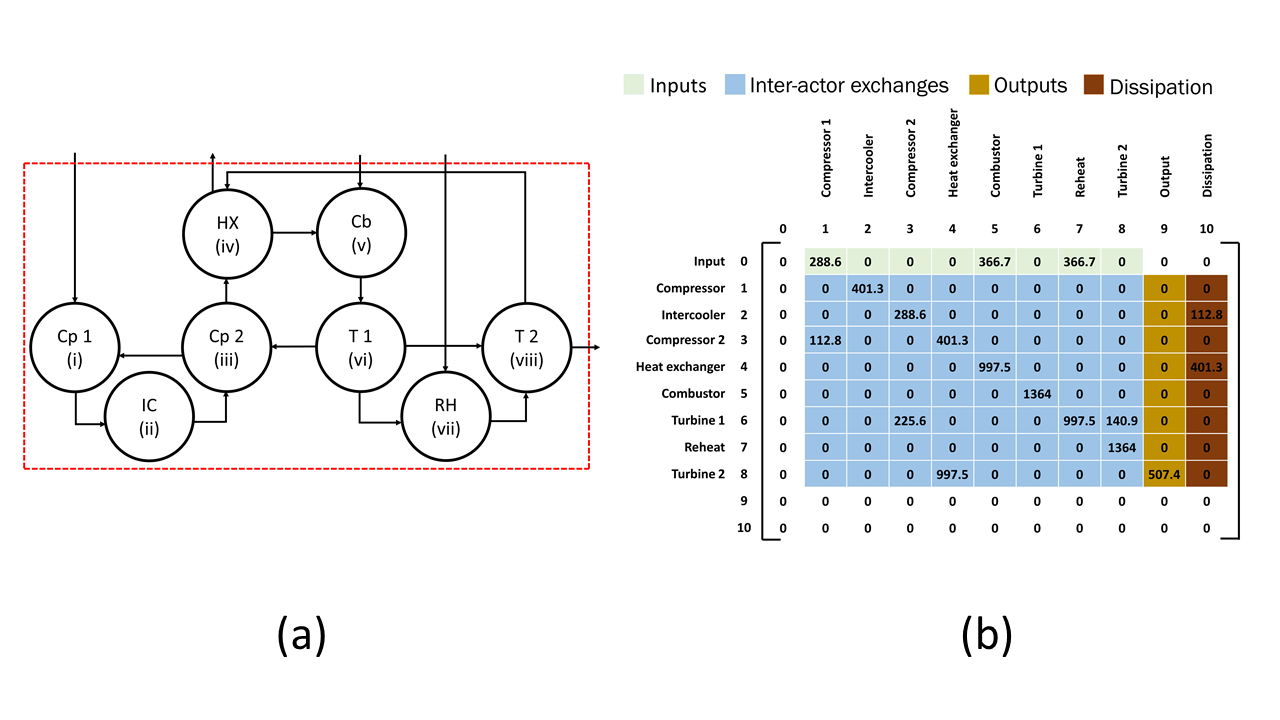

Supplement: S3 Fig — (a) Energy flow diagram, (b) Ecological flow matrix. Cp refers to compressor; HX refers to regeneration heat exchanger; Cb refers to combustor; T refers to turbine. The red dotted square indicates system boundary. (TIF) [file pone.0226993.s003.tif]

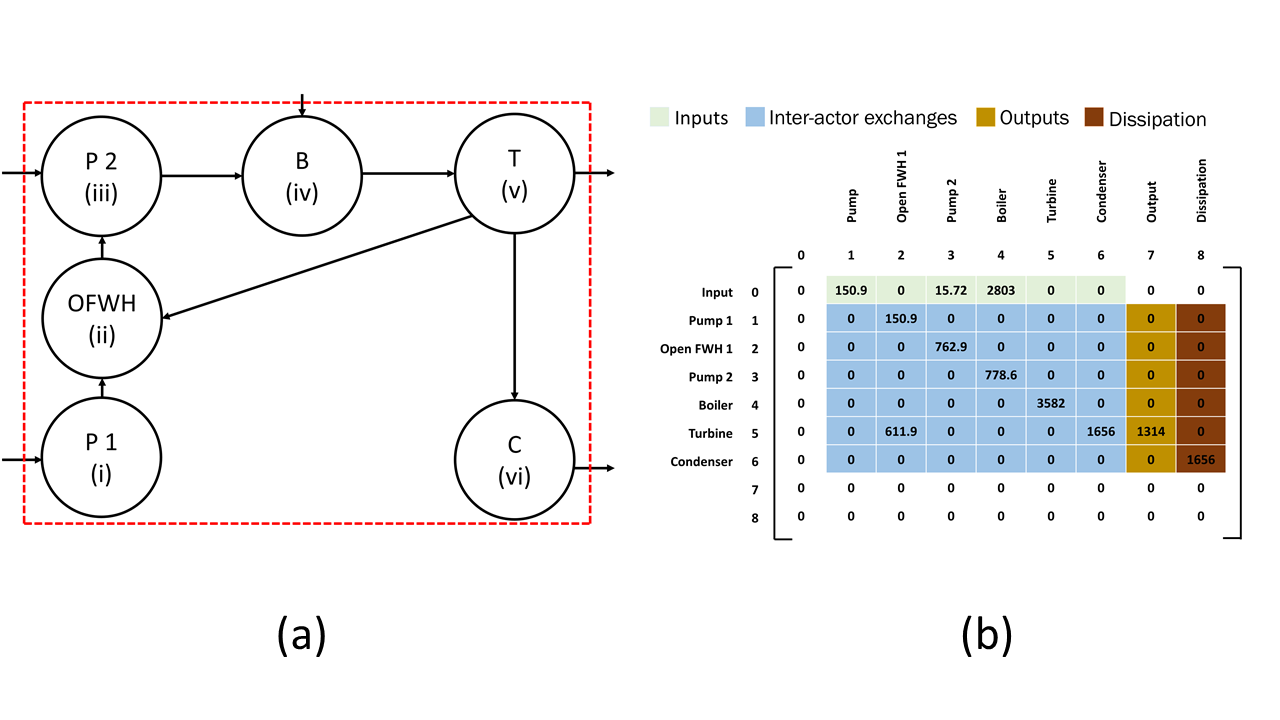

Supplement: S4 Fig — (a) Energy flow diagram, (b) Ecological flow matrix. P refers to pump; OFWH refers to open feed water heater; B refers to boiler; T refers to turbine; C refers to condenser. The red dotted square indicates system boundary. (TIF) [file pone.0226993.s004.tif]

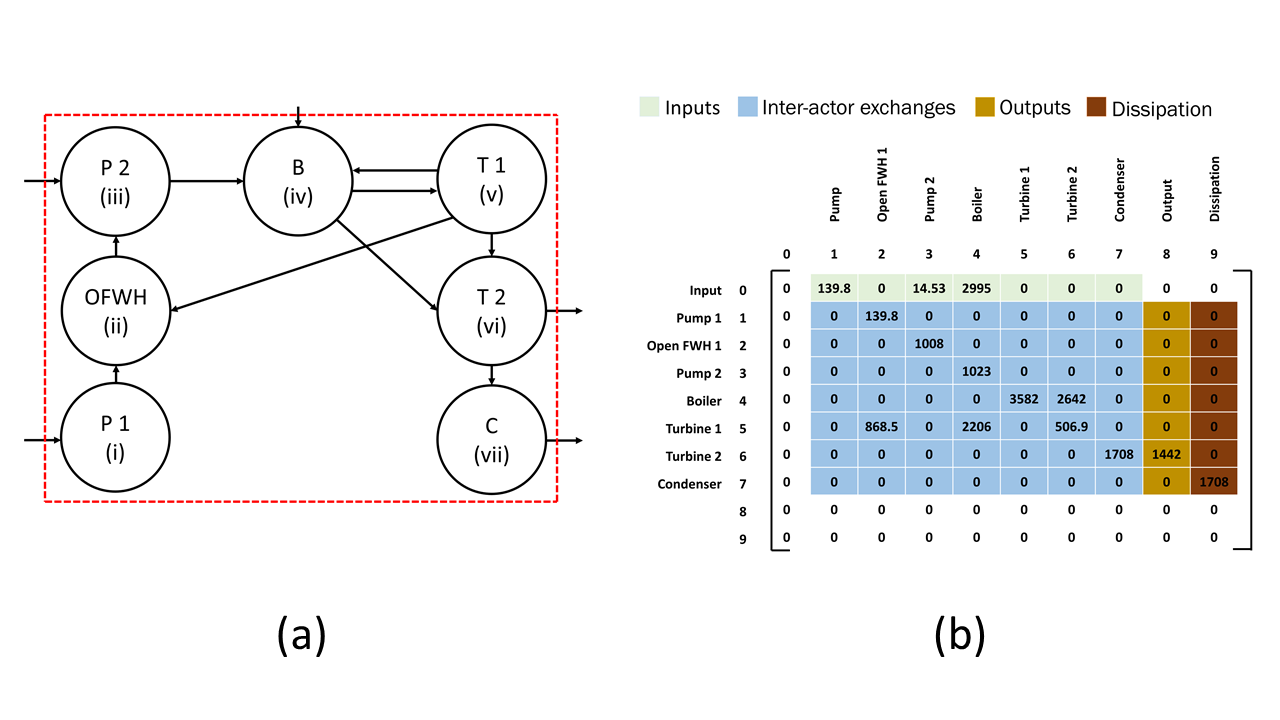

Supplement: S5 Fig — (a) Energy flow diagram, (b) Ecological flow matrix. P refers to pump; OFWH refers to open feed water heater; B refers to boiler; T refers to turbine; C refers to condenser. The red dotted square indicates system boundary. (TIF) [file pone.0226993.s005.tif]
